# Supplementary material for: Mesenchymal stromal cells alleviate depressive and anxiety-like behaviors via a lung vagal-to-brain axis in male mice
Source: Nat Commun. 2023 Nov 16;14:7406. doi: 10.1038/s41467-023-43150-0 (PMC10654509; doi:10.1038/s41467-023-43150-0)
Supplement: Supplementary file 1 — Supplementary Information [file 41467_2023_43150_MOESM1_ESM.pdf]

Supplementary Information

Contents

Supplementary Fig. 1 The effects of MSCs on depressive and anxiety-like behaviors of RSD mice (related to Fig. 1).

Supplementary Fig. 2 The effects of MSC infusion on inflammation level of stressed mice (related to Fig. 1).

Supplementary Fig. 3 The effects of MSCs on the dopaminergic system, hypothalamic-pituitary-adrenal (HPA) axis, and serotonergic system (related to Fig. 1).

Supplementary Fig. 4 5-HT^{DRN} neurons respond positively to MSC administration (related to Fig. 1)

Supplementary Fig. 5 5-HT^{DRN} neurons were eliminated by 5, 7-DHT or AAV injection, and hM4Di-mediated 5-HT+ neuron silencing inhibited the antidepressant effects of MSCs (related to Fig. 2).

Supplementary Fig. 6 The positive response of pulmonary vagal sensory neurons to MSCs in the lungs (related to Fig. 3).

Supplementary Fig. 7 Transsynaptic labeling of PRV-EGFP (related to Fig. 4).

Supplementary Fig. 8 Transcriptional profiling reveals similar features of MSCs before and after transplantation (related to Fig. 5)

Supplementary Fig. 9 The BDNF expression in GFP-MSCs after transplantation (related to Fig. 5).

Supplementary Fig. 10 The role of BDNF in the antidepressant effects of MSCs

(related to Fig. 5).

Supplementary Fig. 11 The intratracheal delivery of 7, 8-DHF alleviated depressive and anxiety-like behaviors, but vagotomy inhibited the antidepressant effects (related to Fig. 6).

Supplementary Fig. 12 The change of 5-HT levels in serum, lung, and ascitic fluid after MSC injection (related to discussion).

Supplementary Fig. 13 The fast-onset antidepressant effects of MSC therapy (related to discussion).

Supplementary Fig. 14 The gating strategy (Fig. 5f).

Supplementary Table 1 Sequence of specific primers used for qRT-PCR analysis.

Supplementary Table 2 Antibodies for immunofluorescence staining.

Supplementary Table 3 Antibodies for flow cytometry.

Supplementary Table 4 Antibodies for western blotting.

Supplementary Table 5 The sequences of RNA interference (RNAi).

Supplementary sequences information The sequences for construction of sLP-mCherry, GFP, and RFP.

Supplementary Fig. 1

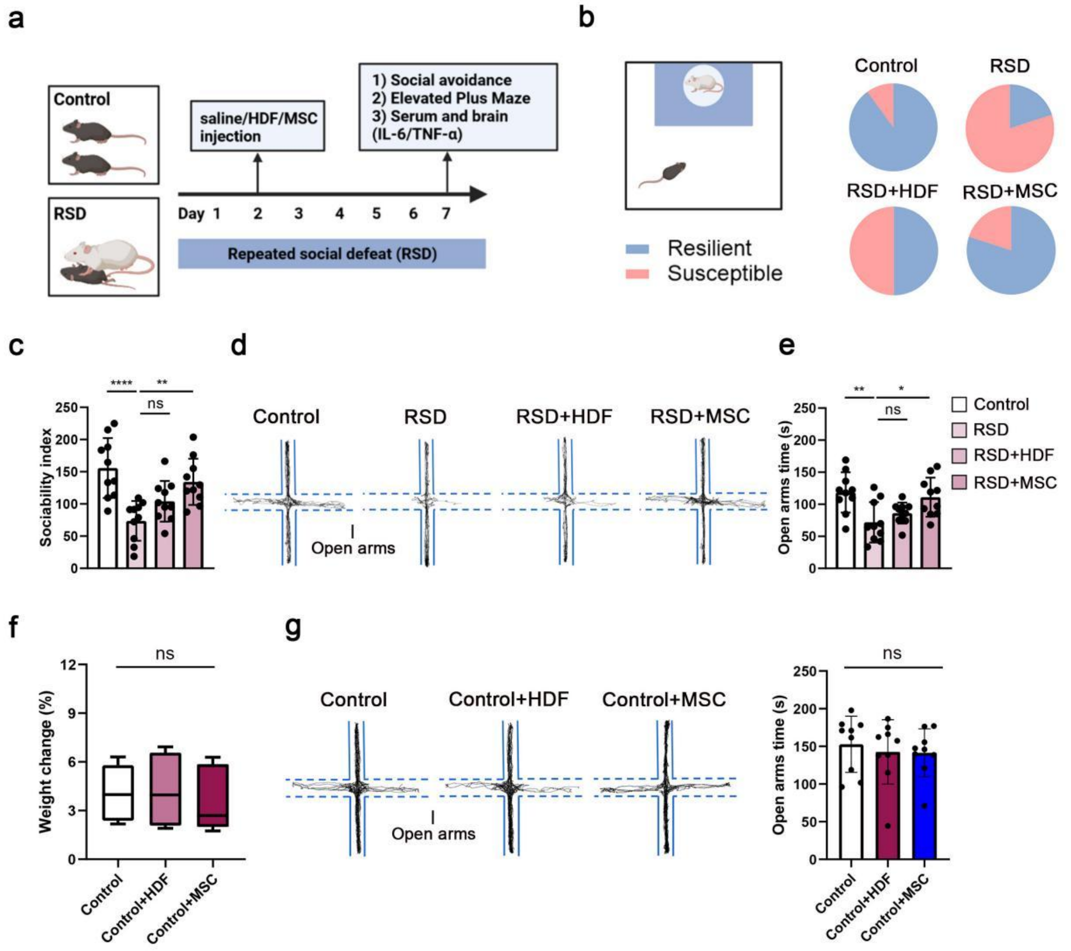

Supplementary Figure 1. The effects of MSCs on depressive and anxiety-like behaviors of RSD mice.

a, Schematic diagram showing the experimental procedures about RSD.

b, Illustration identifying the designated interaction zone used to quantify sociability index (left). Pie charts representing the proportion of animals per group designated as resilient (sociability index scores 100 or above) or susceptible (sociability index scores less than 100).

c, Quantification of sociability index for all groups. One-way ANOVA: $F_{(3, 36)} = 9.477$ **** $P = 0.000094$. n = 10 mice.

d, Representative activity tracking in the EPM.

e, The time spent in the open arms. One-way ANOVA: $F_{(3, 36)} = 5.916$ ** $P = 0.0022$. n = 10 mice.

f, Body weight changes among the four groups after 14 days of CRS. One-way ANOVA: $F_{(2, 12)} = 0.1029$ ns $P = 0.90$. n = 5 mice.

g, Representative activity tracking and the quantification of open arms time in the EPM. One-way ANOVA: $F_{(2, 24)} = 0.2531$ ns $P = 0.78$. n = 9 mice.

Illustrations created with BioRender.com. Source data are provided as a Source Data file.

Supplementary Fig. 2

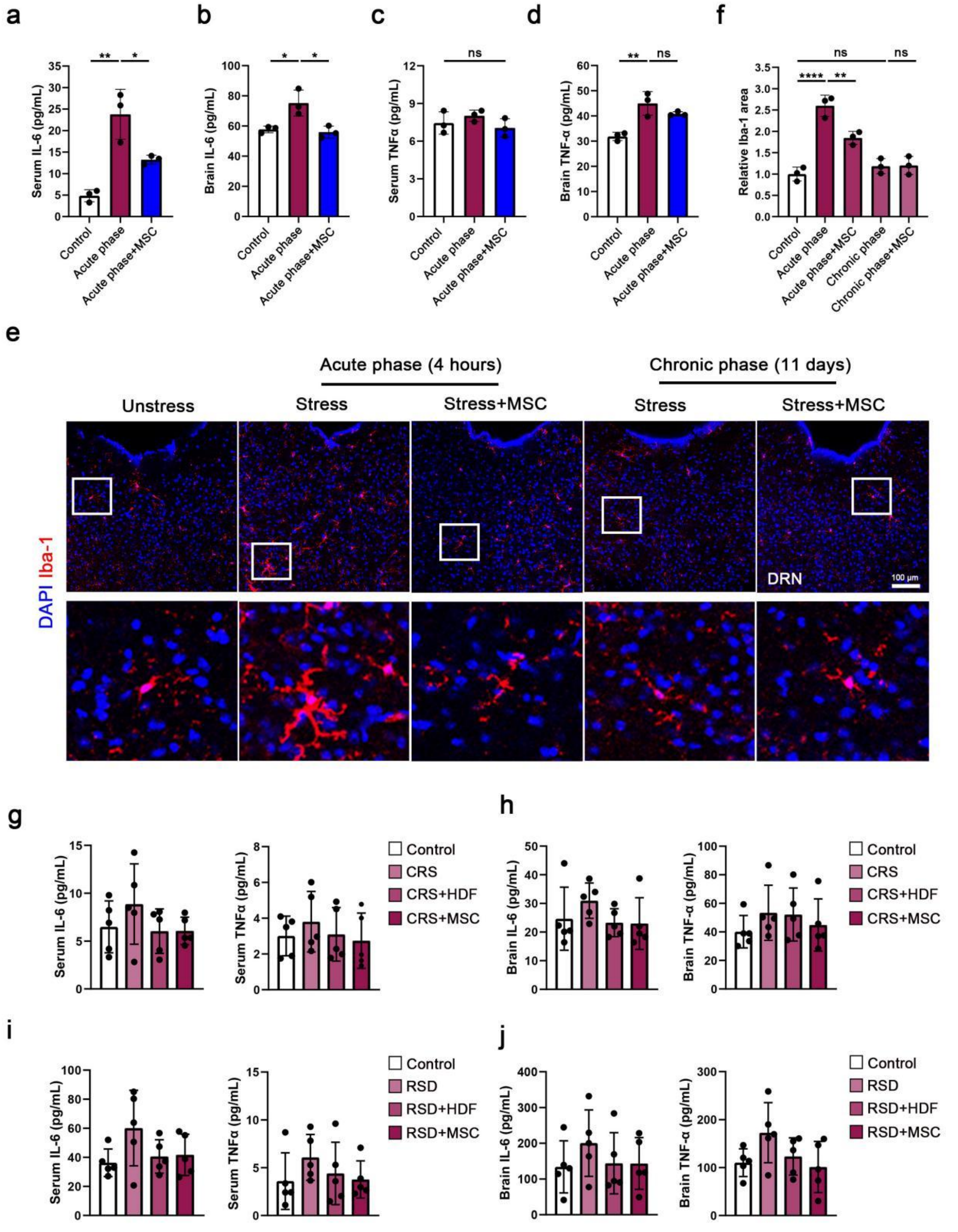

Supplementary Fig. 2 The effects of MSC infusion on inflammation level of acute stress and chronic stress mice.

a. Enzyme-linked immunosorbent assay - quantified serum concentration of interleukin (IL)-6. One-way ANOVA: $F_{(2, 6)} = 21.98$ ** $P = 0.0017$. n = 3 mice.

b. Enzyme-linked immunosorbent assay - quantified brain concentration of interleukin (IL)-6. One-way ANOVA: $F_{(2, 6)} = 10.70$ * $P = 0.0105$. n = 3 mice.

c. Enzyme-linked immunosorbent assay-quantified serum concentration of tumor necrosis factor (TNF)-α. One-way ANOVA: $F_{(2, 6)} = 1.404$ ns $P = 0.3162$. n = 3 mice.

d. Enzyme-linked immunosorbent assay - quantified brain concentration of tumor necrosis factor (TNF)-α. One-way ANOVA: $F_{(2, 6)} = 15.79$ ** $P = 0.0041$. n = 3 mice.

e. Representative images showing positive ionized calcium-binding adapter molecule 1 (Iba-1) immunostaining of microglia in the DRN. Scale bar, 100 µm.

f. Quantification of Iba-1-positive area. One-way ANOVA: $F_{(4, 10)} = 34.70$ **** $P = 0.0000078$. n = 3 mice.

g, i, Enzyme-linked immunosorbent assay - quantified serum concentration of interleukin (IL)-6 and tumor necrosis factor (TNF)-α. Serum IL-6 and TNF-α. n = 5 mice.

h, j, Enzyme-linked immunosorbent assay-quantified brain concentration of interleukin (IL)-6 and tumor necrosis factor (TNF)-α. Brain IL-6 and TNF-α. n = 5 mice.

Source data are provided as a Source Data file.

Supplementary Fig. 3

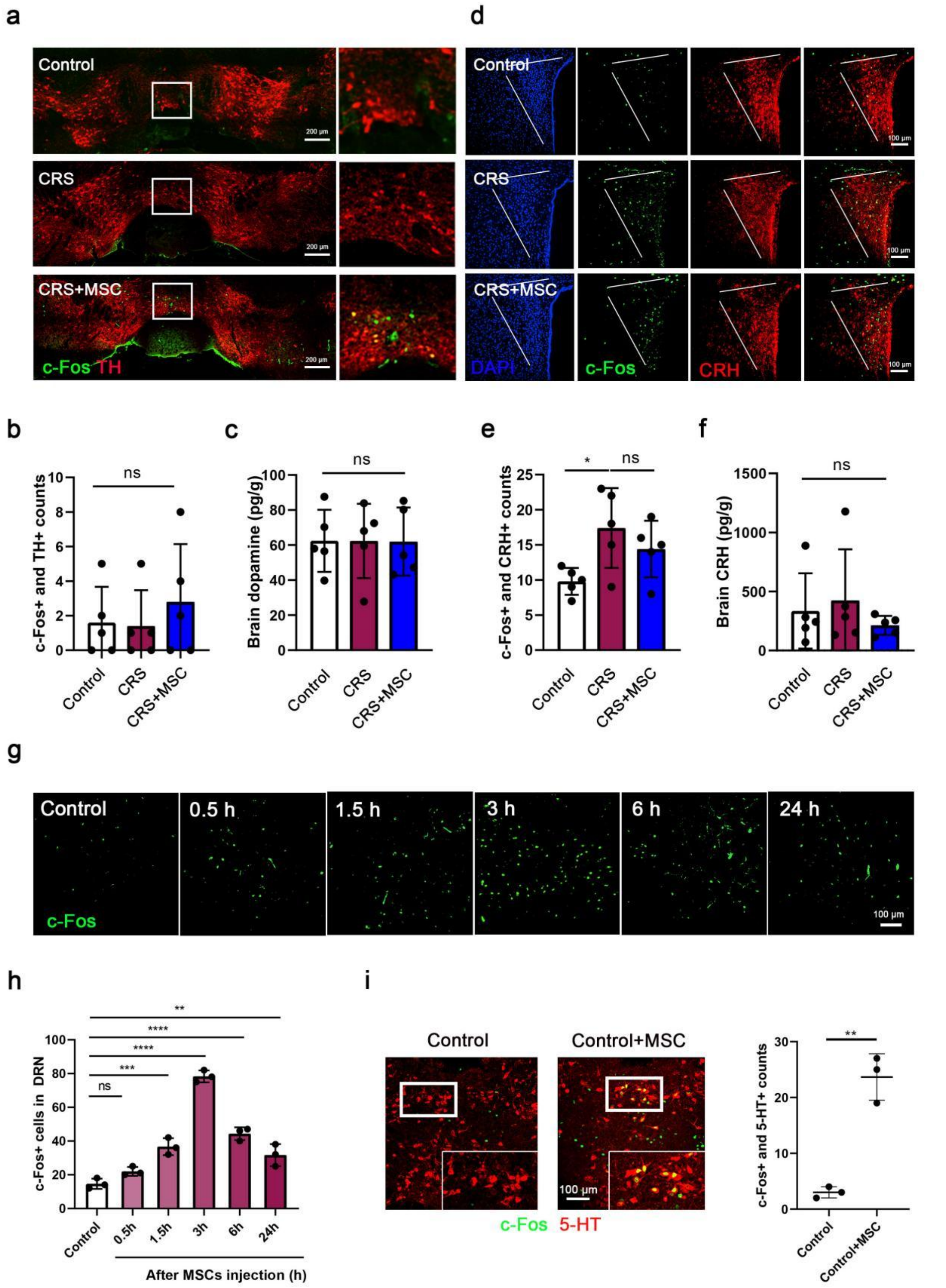

Supplementary Figure 3. The effects of MSCs on the dopaminergic system, hypothalamic-pituitary-adrenal (HPA) axis, and serotonergic system.

a, Representative stained images for co-localization of c-Fos expression (a marker for neuronal activation) and dopamine neurons (TH) in the ventral tegmental area (VTA). Scale bar, 200 μm.

b, Quantification of co-localization between c-Fos and dopamine neurons (TH) in VTA. Kruskal-Wallis test: ns $P = 0.8394$. n = 5 mice.

c, The change of dopamine levels in mice brains detected by ELISA. One-way ANOVA: $F_{(2, 12)} = 0.0005968$ ns $P = 0.9994$. n = 5 mice.

d, Representative stained images for co-localization of c-Fos expression and corticotrophin-releasing hormone (CRH) neurons in the paraventricular nucleus (PVN). Scale bar, 100 μm.

e, Quantification of co-localization between c-Fos and CRH neurons in PVN. One-way ANOVA: $F_{(2, 12)} = 4.203$ * $P = 0.041$. n = 5 mice.

f, The change of CRH levels in mice brains detected by ELISA. Kruskal-Wallis test: ns $P = 0.6808$. n = 5 mice.

g, h, Time course study for investigating c-Fos activation in the DRN for 0.5 - 24 hours following MSC injection. Scale bar, 100 μm. One-way ANOVA: $F_{(5, 12)} = 81.62$ **** $P = 0.0000000077$. n = 3 mice.

i, Representative immunofluorescence images and the quantification showing that 5-hydroxytryptamine (5-HT) and c-Fos co-localization was significantly increased after MSC injection in unstressed mice. Scale bar, 100 μm. Two-tailed t-test: $t = 8.360$, df = 4, Control (n = 3 mice) vs Control + MSC (n = 3 mice) ** $P = 0.0011$.

Source data are provided as a Source Data file.

Supplementary Fig. 4

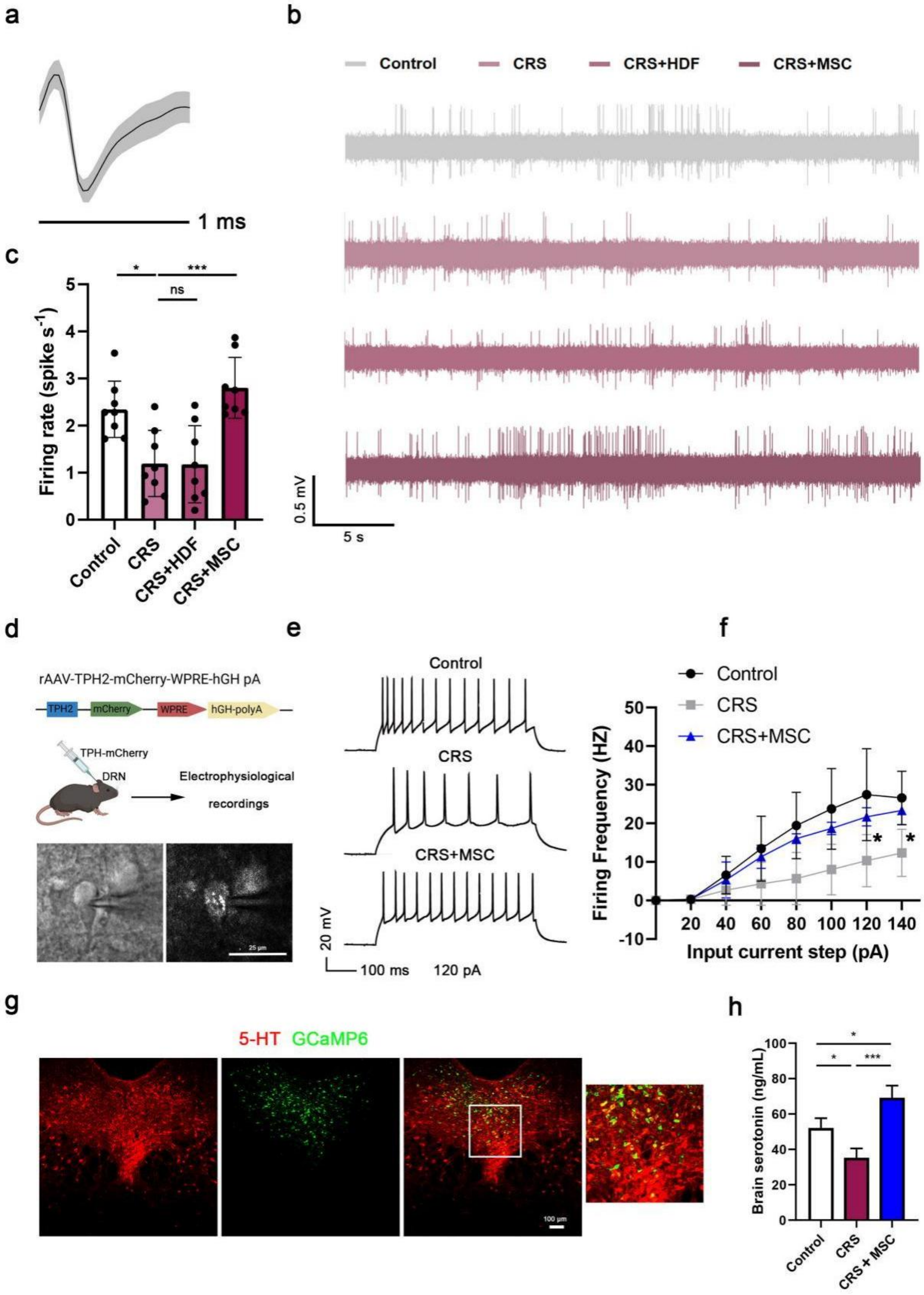

Supplementary Figure 4. 5-HT^{DRN} neurons respond positively to MSC administration

a, One of the spike waveform of 5-HT neuron.

b, Representative trace of electrical activity in DRN neurons of different groups.

c, The Firing rates of the DRN neurons. One-way ANOVA: $F_{(3, 28)} = 11.16$ **** $P = 0.000054$. All groups, n = 8 neurons from three independent experiments.

d, An example of targeted recording of a DRN 5-HT neuron. Visualization of 5-HT neurons is through mice injected with TPH2-mCherry AAV into DRN.

e, Representative action potentials traces in the different groups.

f, The statistics of action potential frequencies in the different groups. 120 pA, Kruskal-Wallis test: ** $P = 0.0016$; Dunn's multiple comparisons test: Control (n = 7) vs CRS (n = 6) ** $P = 0.0067$, CRS (n = 6) vs CRS + MSC (n = 6) * $P = 0.0458$. 140 pA, one-way ANOVA: $F_{(2, 16)} = 10.33$ ** $P = 0.0013$; Tukey's multiple comparisons test: Control (n = 7) vs CRS (n = 6) * $P = 0.0012$, CRS (n = 6) vs CRS + MSC (n = 6) * $P = 0.0126$. The significant statistical differences between CRS and CRS+MSC groups is shown in the line chart. n = several neurons from three mice.

g, Representative immunofluorescence images from three independent experiments showing that GCaMP6 expression selectively in 5-HT neurons. Scale bar, 100 μm.

h, Liquid chromatography with tandem mass spectrometry (LC-MS/MS) was used to quantify the concentration of 5-HT in mice brain tissue. One-way ANOVA: $F_{(2, 6)} = 24.96$ ** $P = 0.0012$. n = 3 mice.

Illustrations created with BioRender.com. Source data are provided as a Source Data file.

Supplementary Fig. 5

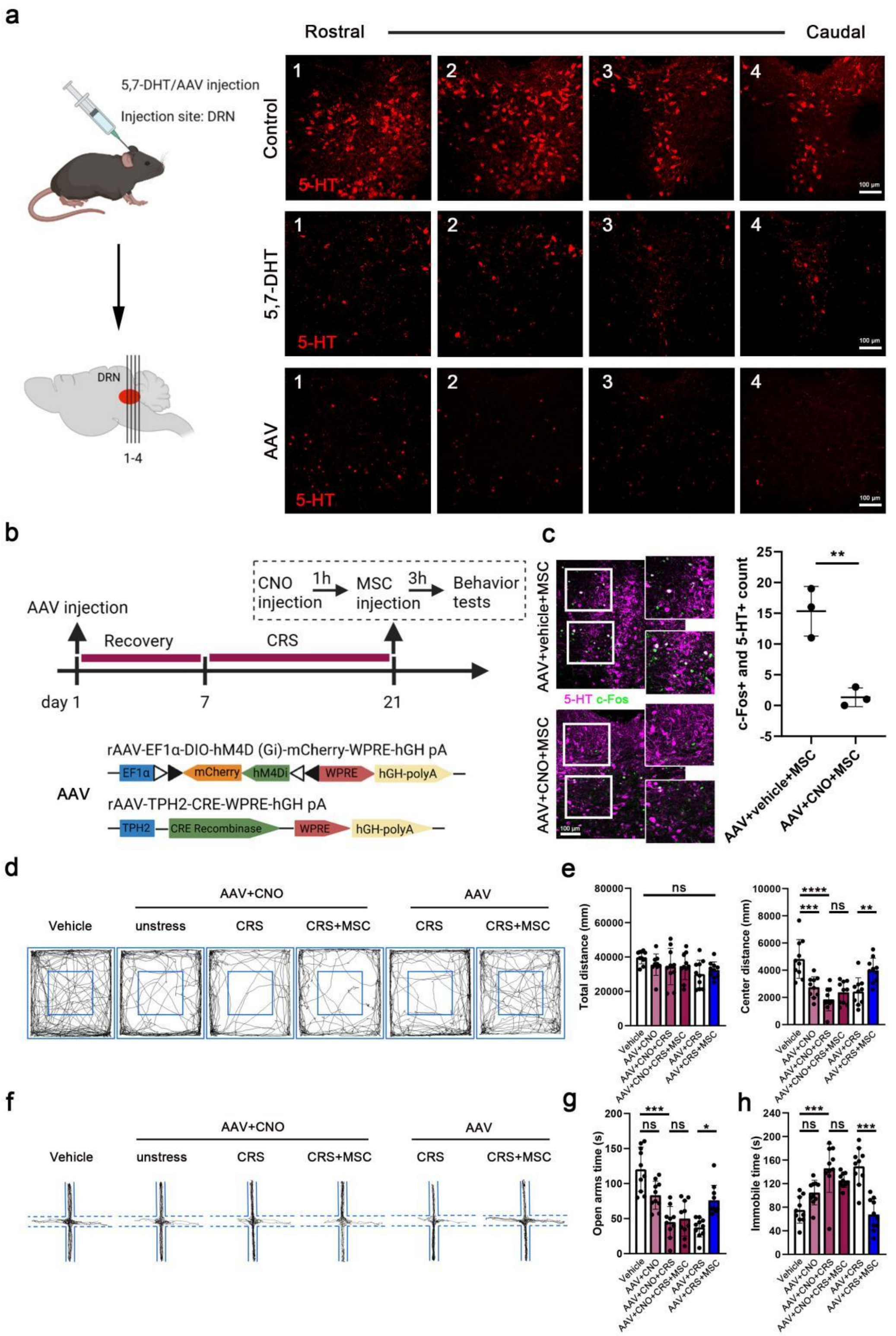

Supplementary Figure 5. 5-HT^{DRN} neurons were eliminated by 5, 7-DHT or AAV injection, and hM4Di-mediated 5-HT+ neuron silencing inhibited the

antidepressant effects of MSC injection

a, Schematic diagram and representative immunofluorescence images showing that the injection of 5,7-DHT or two-virus injection (rAAV-EF1a-DIO-taCasp3 and rAAV-TPH2-CRE) eliminated 5-HT neurons in DRN effectively. Scale bar, 100 μm.

b, Schematic diagram showing the experimental procedures used for hM4Di-mediated 5-HT+ neuron silencing.

c, Representative immunofluorescence images and the quantification showing that hM4Di-mediated 5-HT+ neuron silencing inhibited the increase in c-Fos expression induced by MSC injection. Scale bar, 100 μm. Two-tailed t-test: t = 5.612, df = 4, AAV + Vehicle + MSC (n = 3) vs AAV + CNO + MSC (n = 3) ** P = 0.0050.

d, e, Representative activity tracking and statistical analysis of the total distance and center distance in the OFT. Total distance, Kruskal-Wallis test: ns P = 0.0579. Center distance, one-way ANOVA: $F_{(5, 54)}$ = 13.33 **** P = 0.00000002. n = 10 mice. Mice with AAV and saline (instead of CNO) injection as the Vehicle group.

f, g, Representative activity tracking and statistical analysis of the open arms time in the EPM. Kruskal-Wallis test: **** P = 0.0000004. n = 10 mice.

h, The statistical analysis of the immobile time in the TST. Kruskal-Wallis test: **** P = 0.0000005. n = 10 mice.

Illustrations created with BioRender.com. Source data are provided as a Source Data file.

Supplementary Fig. 6

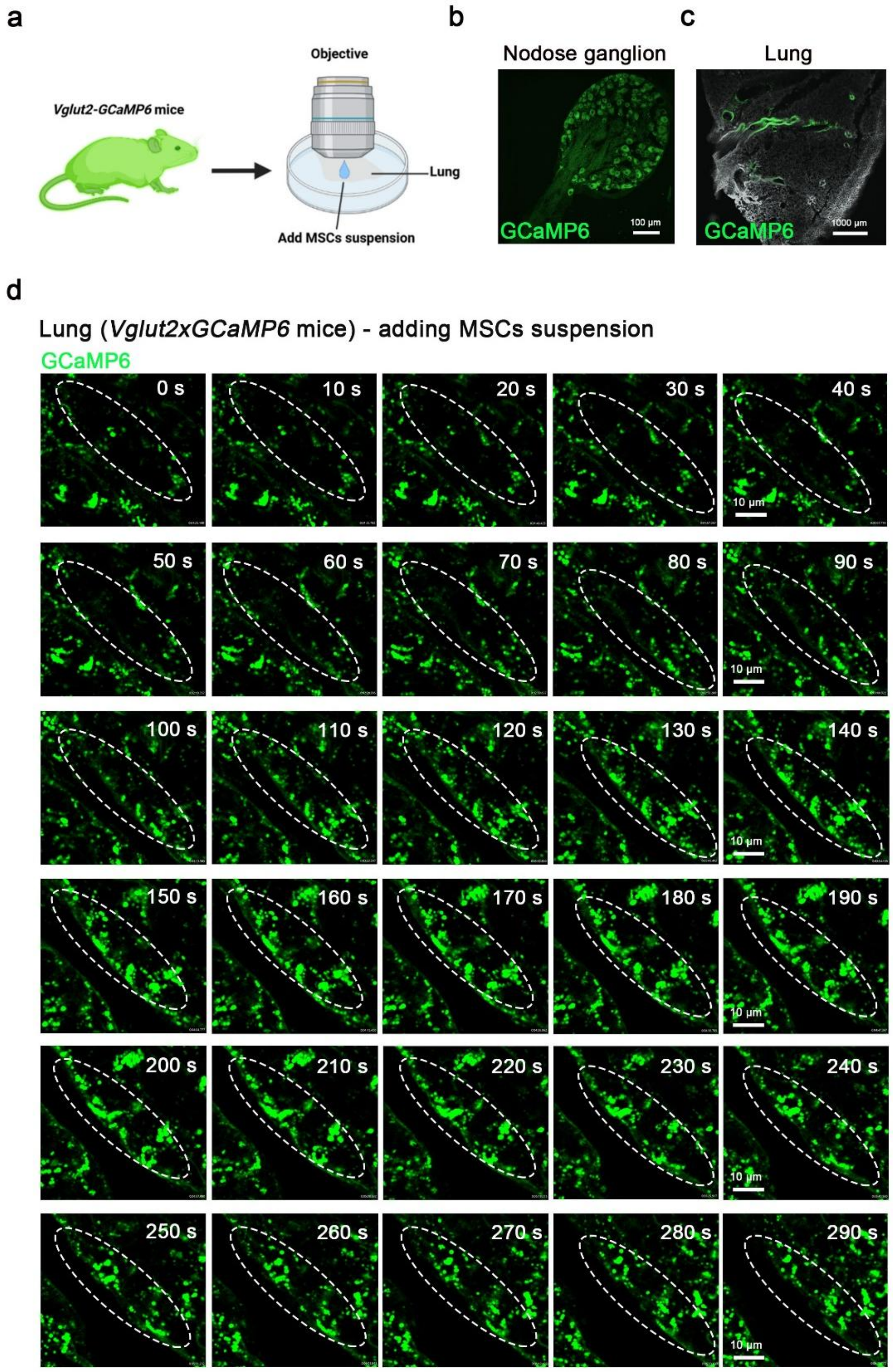

Supplementary Figure 6. The positive response of pulmonary vagal sensory neurons to MSCs in the lungs.

a, Schematic diagram showing the experimental procedures about confocal imaging of ex vivo lungs.

b, Representative images showing that the GCaMP6 fluorescence of the nodose ganglia in *VGLUT2-GCaMP6* mice. Scale bar, 100 μm.

c, Representative images showing that the GCaMP6 fluorescence of the lung in *VGLUT2-GCaMP6* mice. Scale bar, 1000 μm.

d, Sequential images of the pulmonary VGLUT2 innervations displaying Ca^{2+} responses (changes in GCaMP6 fluorescence, green) after adding MSCs suspension of PBS. Scale bar, 10 μm.

Illustrations created with BioRender.com. Source data are provided as a Source Data file.

Supplementary Fig. 7

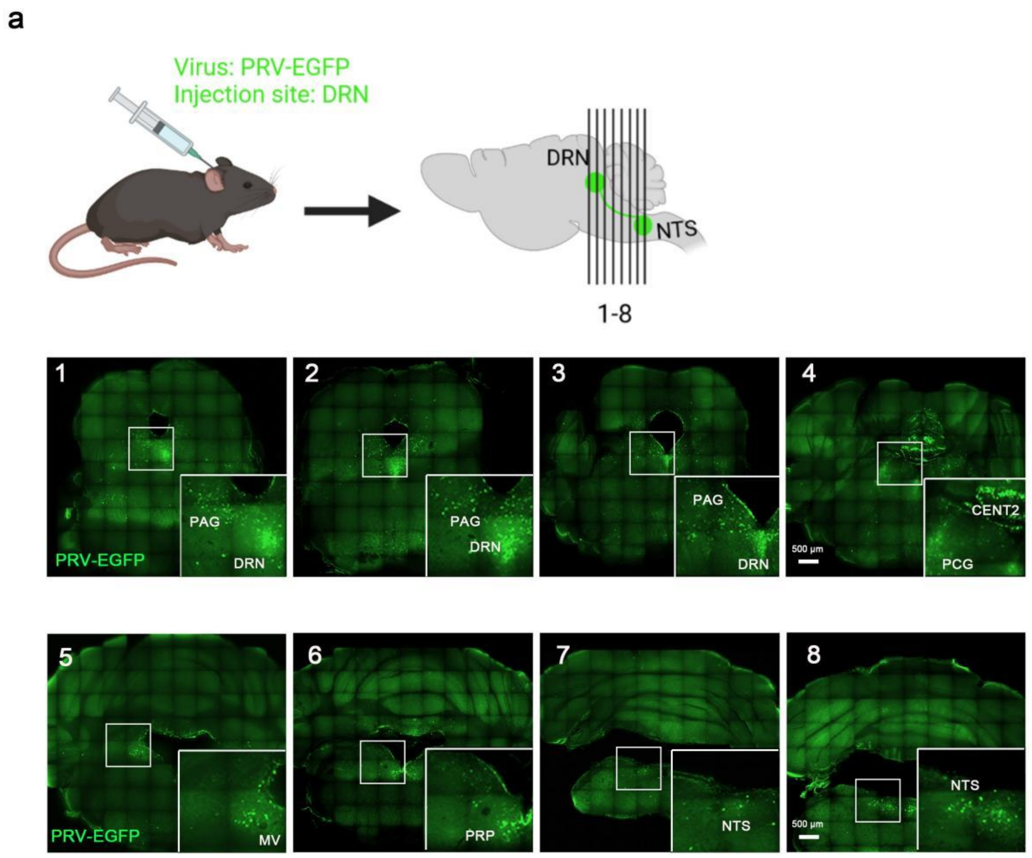

Supplementary Figure 7. Transsynaptic Labeling of PRV-EGFP.

a, Schematic diagram (top). Representative images showing retrograde labeling in brain regions between DRN and NTS. Scale bar, 500 µm. PAG, periaqueductal gray; DRN, dorsal nucleus raphe; CENT2, central lobule II; MV, medial vestibular nucleus; PRP, nucleus prepositus; NTS, nucleus of the solitary tract.

Illustrations created with BioRender.com. Source data are provided as a Source Data file.

Supplementary Fig. 8

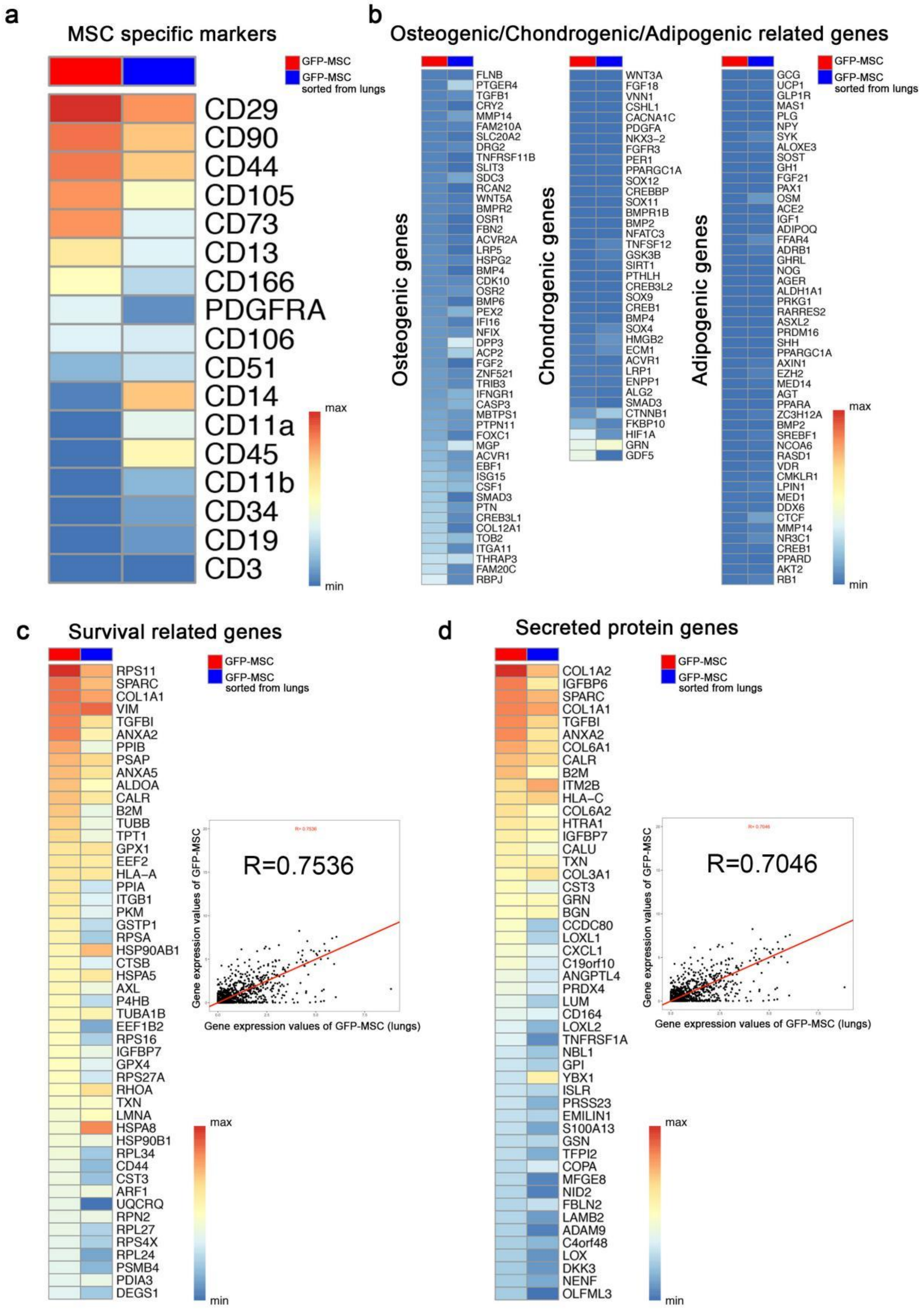

Supplementary Figure 8. Transcriptional profiling reveals similar features of MSCs before and after transplantation.

a, Heat map showing that GFP-MSCs in vitro or GFP-MSCs isolated from lungs had similar expression profiles for MSC-related surface marker-encoding genes.

b, Heat map showing that GFP-MSCs in vitro or GFP-MSCs isolated from lungs were similar with respect to the genes of osteocytes, chondrocytes, and adipocytes, and both were in undifferentiated state.

c, Heat map showing that the two types of GFP-MSCs were very similar in terms of survival genes. (r=0.7536, Pearson correlation coefficient).

d. Heat map showing that the two types of GFP-MSCs were very similar in terms of secretion-related gene. (r=0.7046, Pearson correlation coefficient).

Source data are provided as a Source Data file.

Supplementary Fig. 9

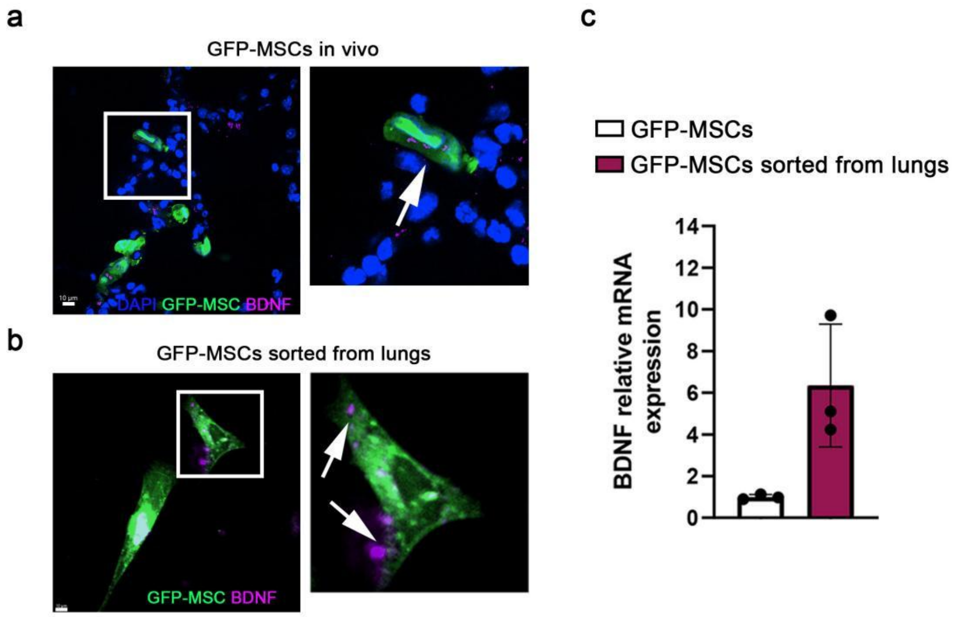

Supplementary Figure 9. The BDNF expression in GFP-MSCs after transplantation.

a, Representative immunofluorescence images showing that the colocalization between the injected GFP-MSCs and BDNF in mice lungs. Scale bar, 10 μm.

b. Representative immunofluorescence images showing that the isolated GFP-MSCs from mice lungs could still secrete BDNF. Scale bar, 10 μm.

c. The qRT-PCR analysis to confirm that the isolated GFP-MSCs from mice lungs could still express BDNF. All groups, n = 3.

Source data are provided as a Source Data file.

Supplementary Fig. 10

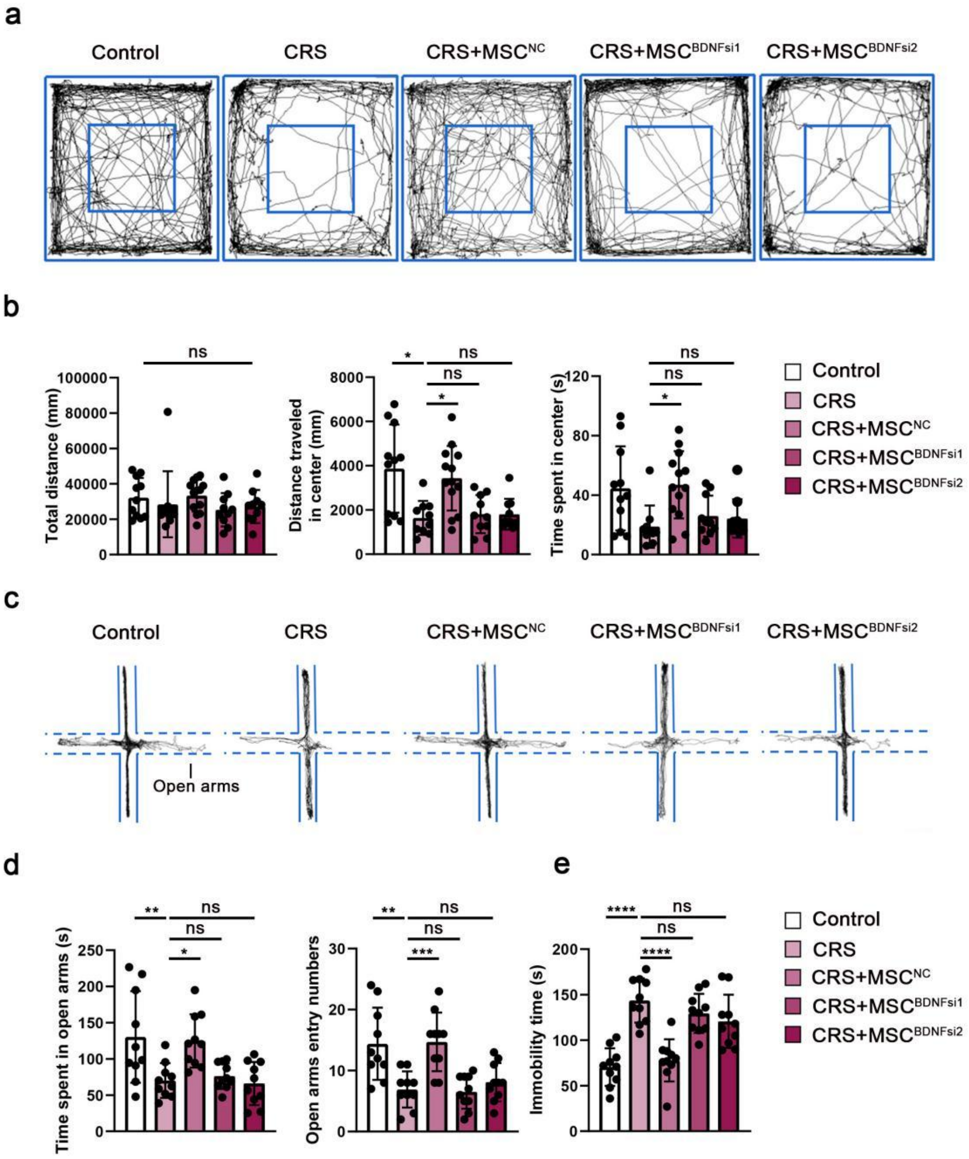

Supplementary Figure 10. The role of BDNF in the antidepressant effects of MSCs.

a, Representative activity tracking in the OFT.

b, The total distance, the distance traveled in the center field, and the time spent in the center field. Total distance, Kruskal-Wallis test: ns P = 0.2002. Center distance, Kruskal-Wallis test: ** P = 0.0016. Center time, Kruskal-Wallis test: * P = 0.0162. n =

11, 10, 13, 10, 10 mice separately.

c, Representative activity tracking in the EPM.

d, The time spent in and entry numbers to the open arms. Open arms time, one-way ANOVA: $F_{(4, 45)} = 6.899$ *** $P = 0.0002$. Open arms entry numbers, one-way ANOVA: $F_{(4, 45)} = 9.885$ **** $P = 0.0000079$. n = 10 mice.

e, The duration of immobility in the TST. One-way ANOVA: $F_{(4, 45)} = 18.35$ **** $P = 0.0000000053$. n = 10 mice.

Source data are provided as a Source Data file.

Supplementary Fig. 11

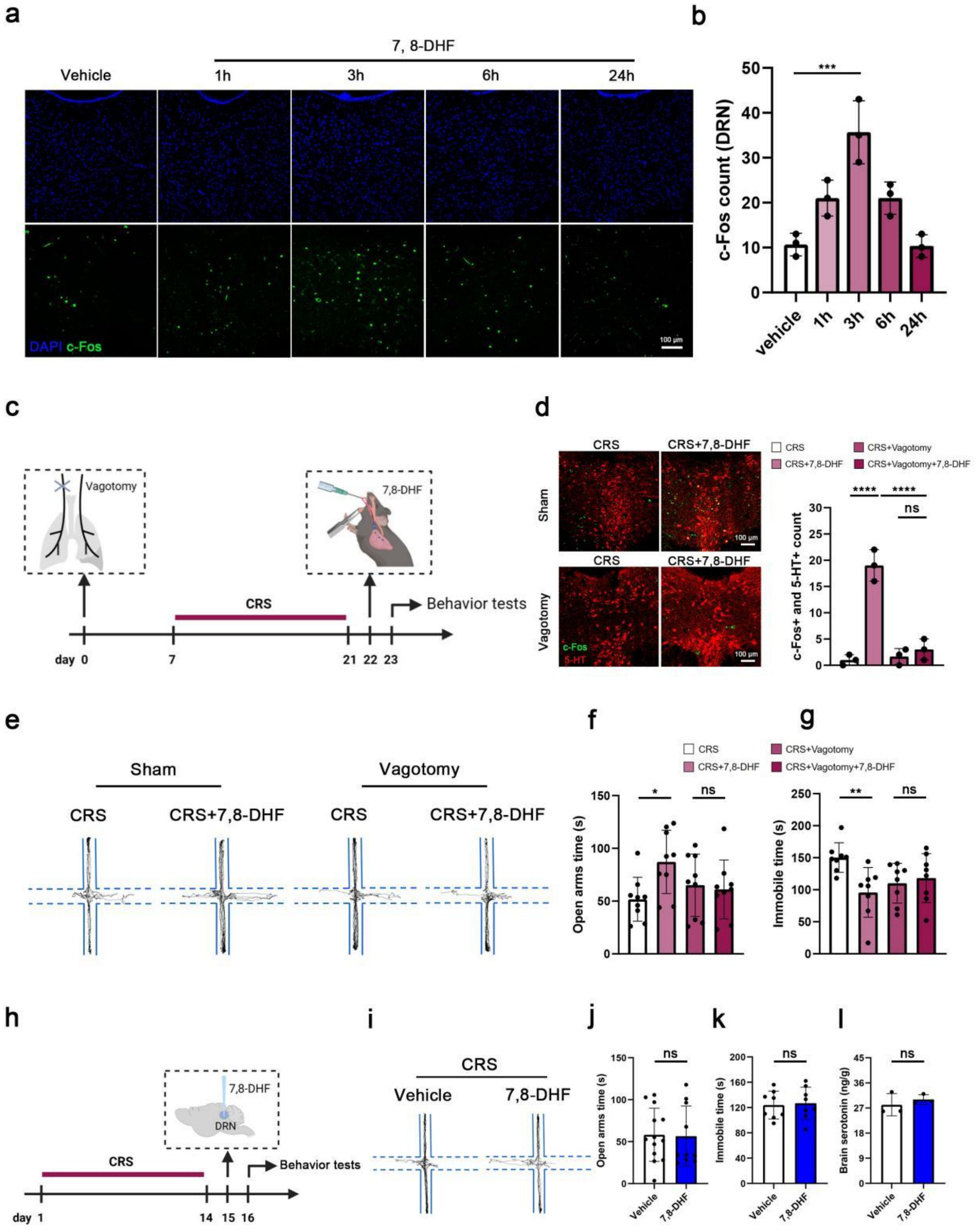

Supplementary Figure. 11 The intratracheal delivery of 7, 8-DHF alleviated depressive and anxiety-like behaviors, but vagotomy inhibited the antidepressant

effects.

a, Time course study for investigating c-Fos expression in the DRN for 1 - 24 hours following the intratracheal infusion of 7, 8-DHF. Scale bar, 100 μm.

b, The quantification of DRN c-Fos expression. One-way ANOVA: $F_{(4, 10)} = 17.62$ *** $P = 0.0002$. n = 3 mice.

c, Schematic diagram showing the experimental procedures.

d, Representative images and the quantification showing that vagotomy reduced the co-localization between 5-HT and c-Fos in DRN induced by the intratracheal infusion of 7, 8-DHF. Scale bar, 100 μm. One-way ANOVA: $F_{(3, 8)} = 56.02$ **** $P = 0.000010$. n = 3 mice.

e, The representative activity tracks of EPM.

f, The statistical analysis of EPM. Two-tailed t-test: $t = 2.911$, df = 16, CRS vs CRS + 7, 8-DHF * $P = 0.0102$. Two-tailed t-test: $t = 0.2926$, df = 16, CRS + Vagotomy vs CRS + Vagotomy + 7, 8-DHF ns $P = 0.7736$. n = 9 mice.

g, The statistical analysis of the immobile time in the TST. Two-tailed t-test: $t = 3.409$, df = 14, CRS vs CRS+7, 8-DHF ** $P = 0.0042$. Two-tailed t-test: $t = 0.4671$, df = 14, CRS + Vagotomy vs CRS + Vagotomy + 7, 8-DHF ns $P = 0.6476$. n = 8 mice.

h, Schematic diagram showing the experimental procedures.

i, The representative activity tracks of EPM.

j, The statistical analysis of EPM. Two-tailed t-test: $t = 0.1275$, df=21, CRS + Vehicle (n = 13 mice) vs CRS + 7, 8-DHF (n = 10 mice) ns $P = 0.8997$.

k, The statistical analysis of the immobile time. Two-tailed t-test: $t = 0.2411$, df = 14.

CRS + Vehicle vs CRS + 7, 8-DHF ns P = 0.8130. n = 8 mice

l, The brain 5-HT level. Two-tailed t-test: t = 0.7543, df = 4, CRS + Vehicle vs CRS + 7, 8-DHF ns P = 0.4926. n = 3 mice.

Illustrations created with BioRender.com. Source data are provided as a Source Data file.

Supplementary Fig. 12

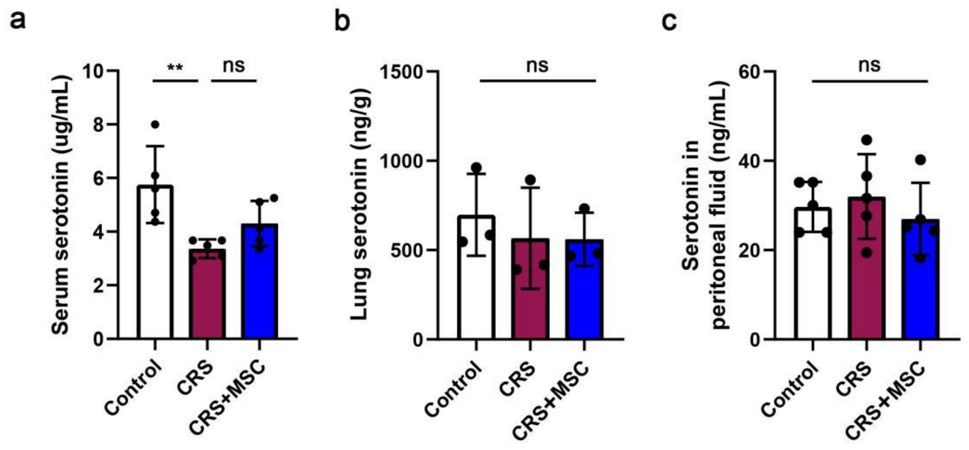

Supplementary Fig 12. The change of 5-HT levels in serum, lung, and ascitic fluid after MSC injection.

a, The change of 5-HT levels in serum after MSC injection. One-way ANOVA: F (2, 12) = 7.544 *** P = 0.0076. All groups, n = 5 mice.

b, The change of 5-HT levels in lung tissue homogenate after MSC injection. One-way ANOVA: $F_{(2, 6)}$ = 0.3487 ns P = 0.7190. All groups, n = 3 mice.

c, The change of 5-HT levels in ascitic fluid after MSC injection. One-way ANOVA: $F_{(2, 12)}$ = 0.5085 ns P = 0.6138. All groups, n = 5 mice.

Supplementary Fig. 13

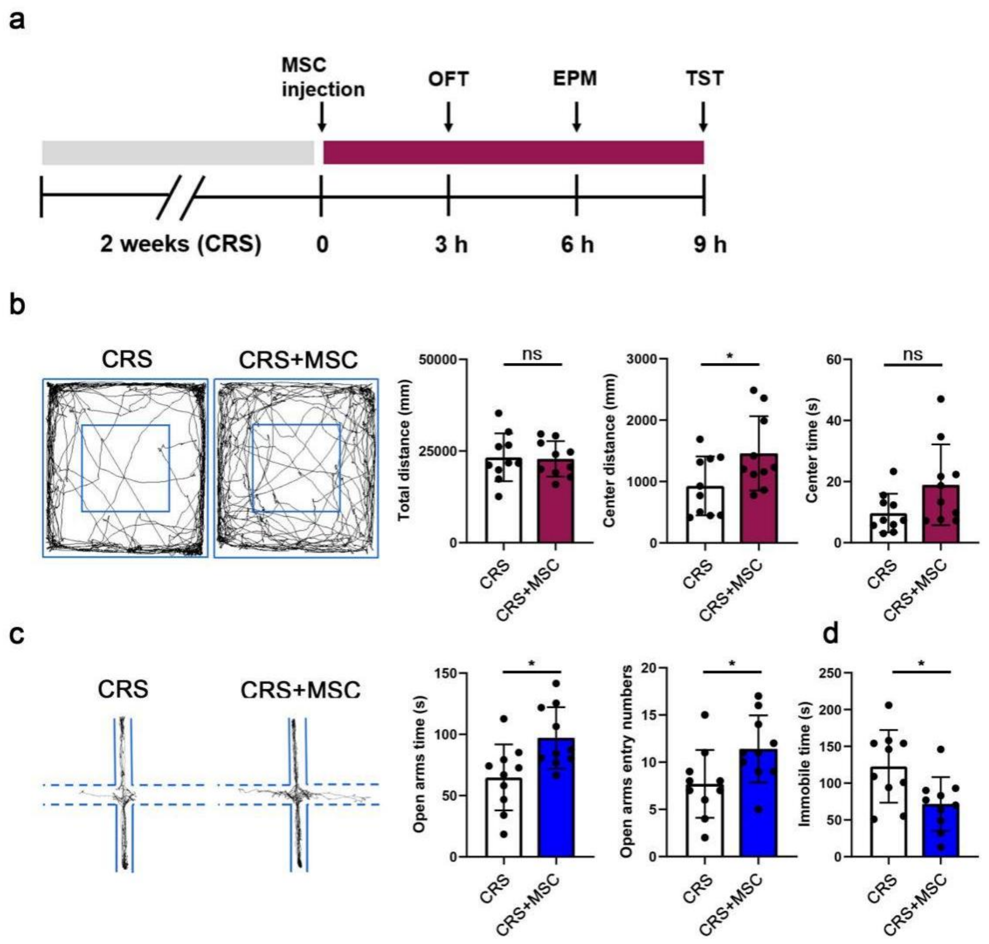

Supplementary Fig 13. The fast-onset antidepressant effects of MSC therapy.

a, Schematic diagram showing the experimental procedures about MSC treatment in chronic restraint stress (CRS) mice.

b, Representative activity tracking in the OFT, and the total distance, the distance traveled in the center field (center distance), and the time spent in the center field (center time). Total distance, Two-tailed t-test: t = 0.1707, df = 18, CRS vs CRS + MSC ns P = 0.8664. Center distance, Two-tailed t-test: t = 2.174, df = 18, CRS vs CRS + MSC * P = 0.0433. Center time, Two-tailed t-test: t = 1.991, df = 18, CRS vs CRS + MSC ns P = 0.0619. All groups, n = 10 mice.

c, Representative activity tracking in the EPM, and the time spent in (open arms time)

and entry numbers to the open arms (open arms entry numbers). Open arms time, Two-tailed t-test: $t = 2.774$, df = 18 CRS vs CRS + MSC * $P = 0.0125$. Open arms entry numbers, Two-tailed t-test: $t = 2.312$, df = 18, CRS vs CRS + MSC * $P = 0.0328$. All groups, n = 10 mice.

d, The duration of immobility in the TST. Two-tailed t-test: $t = 2.631$, df = 18, CRS vs CRS + MSC * $P = 0.0170$. All groups, n = 10 mice.

Supplementary Fig. 14

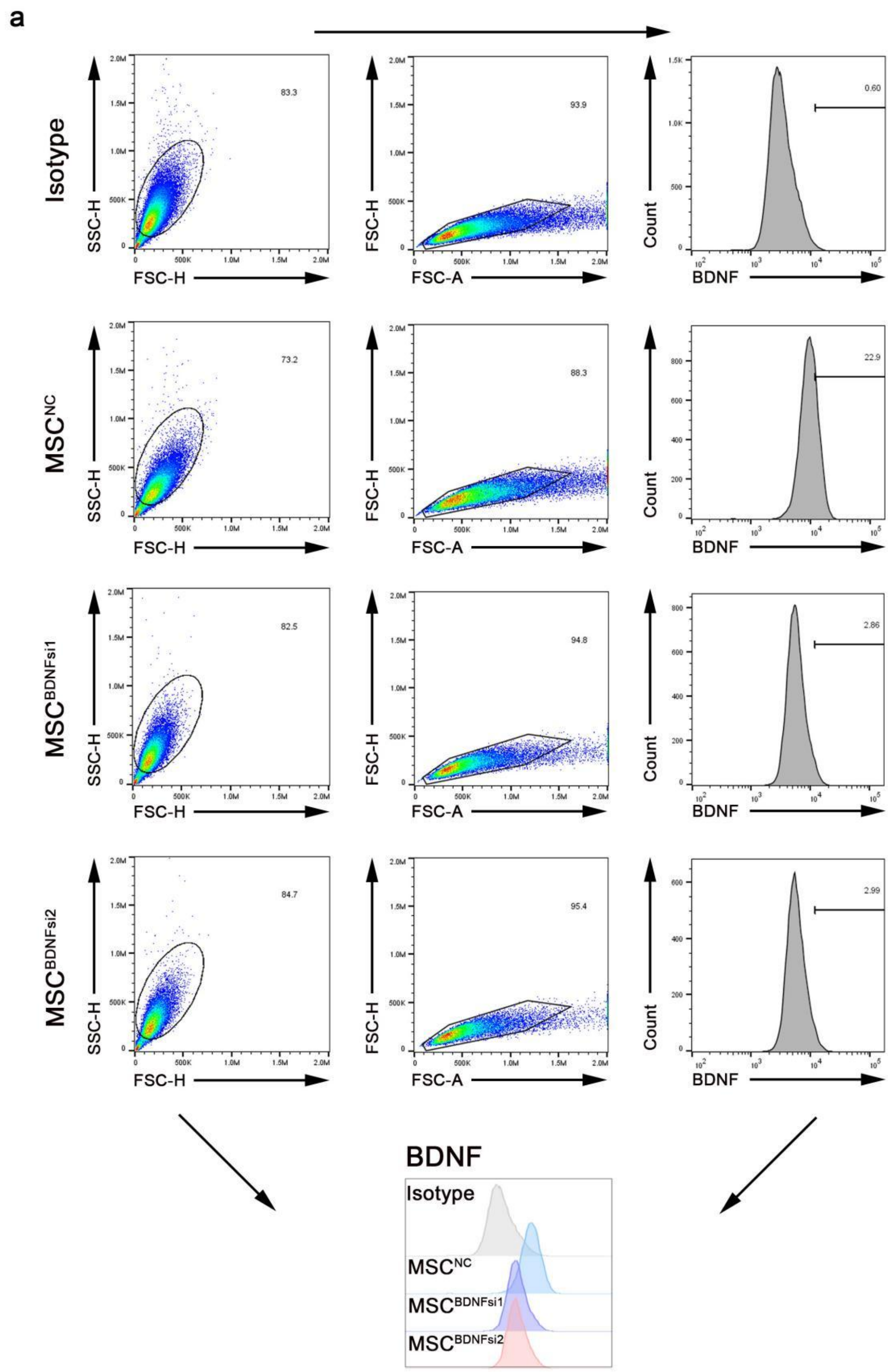

Supplementary Fig 14. The gating strategy (Fig. 5f).

a, The gating strategy of Fig 5f.

Supplementary Table 1. Sequence of specific primers used for qRT-PCR analysis

Genes	Forward Sequence (5'-3')	Reverse Sequence (5'-3')
Human GAPDH	GAA GGT GAA GGT CGG AGT C	GAA GAT GGT GAT GGG ATT TC
Human BDNF	CATCCGAGGACAAGGTGGCTTG	GCCGAACTTTCTGGTCCTCATC
Human GDNF	CGCCGAAGACCGCTCCCTCG	ATCCATGACATCATCGAACTGATC
Human NGF	ACCCGCAACATTACTGTGGACC	GACCTCGAAGTCCAGATCCTGA
Mouce GAPDH	CTGAGTTCGTGGAGTCTACTGG	GTCATATTTCTCGTGGTTCACACC
Mouce c-Fos	AGCGAGCAACTGAGAAGACT	GCGTTGAAACCCGAGAACAT

Supplementary Table 2. Antibodies for immunofluorescence staining

Antigen	Source	Dilution	Identifier
Anti-c-Fos	Abcam	1:1000	ab208942
Anti-5-HT	Immunostar	1:500	20080
Anti-Iba-1	FUJIFILM Wako Pure Chemical Corporation	1:1000	019-19741
Anti-TH	Millipore	1:1000	AB152

Anti-CRH	Immunostar	1:500	20084
Anti-Tubb3	Abcam	1:500	ab18207
Anti-VGLUT2	Abcam	1:300	ab216463
Anti-Phospho-TrkA+TrkB	Bioss	1:250	bs-3457R
Goat Anti-mouse IgG Alexa 488	Invitrogen	1:1000	A-11001
Goat Anti-Rabbit IgG Alexa 488	Invitrogen	1:1000	A-11008
Goat Anti-Rabbit IgG Alexa 594	Invitrogen	1:1000	A-11012
Goat Anti-Rabbit IgG Alexa 647	Invitrogen	1:1000	A-21245

Supplementary Table 3. Antibodies for flow cytometry

Antigen	Source	Dilution	Identifier
Anti-BDNF	Abcam	1:30	ab108319
Goat Anti-Rabbit IgG Alexa 594	Invitrogen	1:1000	A-11012

Supplementary Table 4. Antibodies for western blotting

Antigen	Source	Dilution	Identifier
Anti-c-Fos	Abcam	1:1000	ab208942
Anti-BDNF	Abcam	1:1000	ab108319
Anti-TrkB	CST	1:1000	4603S
Anti-Phospho-TrkA+TrkB	Bioss	1:1000	bs-3457R
Anti-GAPDH	CST	1:2000	2118S
Anti-rabbit IgG, HRP-linked Antibody	CST	1:2000	7074S

Anti-mouse IgG, HRP-linked Antibody	CST	1:2000	7076S

Supplementary Table 5. The sequences of RNA interference (RNAi)

Genes	Sequence (5'-3')
BDNF si1	CGGTCACAGTCCTTGAAAA
BDNF si2	CCCTTACCATGGATAGCAA

Supplementary sequences information

The sequences for construction of sLP-mCherry, GFP, and RFP.

1. The sLP-mCherry sequence 5' - 3'

GGATCCATGTGGTGGCGCCTGTGGTGGCTGCTGCTGCTGCTGCTGCTG

TGGCCCATGGTGTGGGCCaagcttTACGCCCGCAAGGCCGCCCGCCAGGCCC

GCGCCGATCCACCGGTCGCCACCATGGTGAGCAAGGGCGAGGAGGATAA

CATGGCCATCATCAAGGAGTTCATGCGCTTCAAGGTGCACATGGAGGGCT

CCGTGAACGGCCACGAGTTCGAGATCGAGGGCGAGGGCGAGGGCCGCCC

CTACGAGGGCACCCAGACCGCCAAGCTGAAGGTGACCAAGGGTGGCCCC

CTGCCCTTCGCCTGGGACATCCTGTCCCCTCAGTTCATGTACGGCTCCAAG

GCCTACGTGAAGCACCCCGCCGACATCCCCGACTACTTGAAGCTGTCCTTC

CCCGAGGGCTTCAAGTGGGAGCGCGTGATGAACTTCGAGGACGGCGGCGT

GGTGACCGTGACCCAGGACTCCTCCCTGCAGGACGGCGAGTTCATCTACA

AGGTGAAGCTGCGCGGCACCAACTTCCCCTCCGACGGCCCCGTAATGCAG

AAGAAGACCATGGGCTGGGAGGCCTCCTCCGAGCGGATGTACCCCGAGG

ACGGCGCCCTGAAGGGCGAGATCAAGCAGAGGCTGAAGCTGAAGGACGG

CGGCCACTACGACGCTGAGGTCAAGACCACCTACAAGGCCAAGAAGCCC

GTGCAGCTGCCCGGCGCCTACAACGTCAACATCAAGTTGGACATCACCTC

CCACAACGAGGACTACACCATCGTGGAACAGTACGAACGCGCCGAGGGC

CGCCACTCCACCGGCGGCATGGACGAGCTGTACAAGTCCGGACTCAGATC

TCGACCGGGCACCATGGCCCCTAAGAAGAAGAGGAAGGTGGAGGCGTTG

CTTCGAATTCTGCAGTCGACGCAAGCTGGGGATCTCGAGCAGAAGCTGAT

CAGCGAGGAGGACCTGTAA

2. The GFP sequence 5’ - 3’

ATGGTGAGCAAGGGCGAGGAGCTGTTCACCGGGGTGGTGCCCATCCTGGT

CGAGCTGGACGGCGACGTAAACGGCCACAAGTTCAGCGTGTCCGGCGAG

GGCGAGGGCGATGCCACCTACGGCAAGCTGACCCTGAAGTTCATCTGCAC

CACCGGCAAGCTGCCCGTGCCCTGGCCCACCCTCGTGACCACCCTGACCT

ACGGCGTGCAGTGCTTCAGCCGCTACCCCGACCACATGAAGCAGCACGAC

TTCTTCAAGTCCGCCATGCCCGAAGGCTACGTCCAGGAGCGCACCATCTTC

TTCAAGGACGACGGCAACTACAAGACCCGCGCCGAGGTGAAGTTCGAGG

GCGACACCCTGGTGAACCGCATCGAGCTGAAGGGCATCGACTTCAAGGAG

GACGGCAACATCCTGGGGCACAAGCTGGAGTACAACTACAACAGCCACA

ACGTCTATATCATGGCCGACAAGCAGAAGAACGGCATCAAGGTGAACTTC

AAGATCCGCCACAACATCGAGGACGGCAGCGTGCAGCTCGCCGACCACTA

CCAGCAGAACACCCCCATCGGCGACGGCCCCGTGCTGCTGCCCGACAACC

ACTACCTGAGCACCCAGTCCGCCCTGAGCAAAGACCCCAACGAGAAGCGC

GATCACATGGTCCTGCTGGAGTTCGTGACCGCCGCCGGGATCACTCTCGG

CATGGACGAGCTGTACAAGTAA

3. The RFP sequence 5’ - 3’

ATGGATAGCACTGAGAACGTCATCAAGCCCTTCATGCGCTTCAAGGTGCA

CATGGAGGGCTCCGTGAACGGCCACGAGTTCGAGATCGAGGGCGAGGGC

GAGGGCAAGCCCTACGAGGGCACCCAGACCGCCAAGCTGCAGGTGACCA

AGGGCGGCCCCCTGCCCTTCGCCTGGGACATCCTGTCCCCCCAGTTCCAGT

ACGGCTCCAAGGTGTACGTGAAGCACCCCGCCGACATCCCCGACTACAAG

AAGCTGTCCTTCCCCGAGGGCTTCAAGTGGGAGCGCGTGATGAACTTCGA

GGACGGCGGCGTGGTGACCGTGACCCAGGACTCCTCCCTGCAGGACGGCA

CCTTCATCTACCACGTGAAGTTCATCGGCGTGAACTTCCCCTCCGACGGCC

CCGTAATGCAGAAGAAGACTCTGGGCTGGGAGCCCTCCACCGAGCGCCTG

TACCCCCGCGACGGCGTGCTGAAGGGCGAGATCCACAAGGCGCTGAAGCT

GAAGGGCGGCGGCCACTACCTGGTGGAGTTCAAGTCAATCTACATGGCCA

AGAAGCCCGTGAAGCTGCCCGGCTACTACTACGTGGACTCCAAGCTGGAC

ATCACCTCCCACAACGAGGACTACACCGTGGTGGAGCAGTACGAGCGCGC

CGAGGCCCGCCACCACCTGTTCCAGTAG
